# The Prevalence of E-Gambling and of Problem E-Gambling in Poland

**DOI:** 10.3390/ijerph17020404

**Published:** 2020-01-08

**Authors:** Bernadeta Lelonek-Kuleta, Rafał P. Bartczuk, Michał Wiechetek, Joanna Chwaszcz, Iwona Niewiadomska

**Affiliations:** Institute of Psychology, The John Paul II Catholic University of Lublin, 20-950 Lublin, Poland; bartczuk@kul.lublin.pl (R.P.B.); wiechetek@kul.lublin.pl (M.W.); chwaszcz@kul.lublin.pl (J.C.); pfrau@kul.lublin.pl (I.N.)

**Keywords:** e-gambling, e-gambling prevalence, forms of e-gambling, problem e-gambling

## Abstract

This study estimated the levels of involvement in e-gambling and problem e-gambling in Poland and identified selected sociodemographic variables associated with e-gambling activities. The study was conducted using a representative sample of the adult inhabitants of Poland (*n* = 2000). The survey contained questions measuring three aspects of gambling (involvement in e-gambling, types of e-gambling activity, and problematic e-gambling). Results suggested that 4.1% of respondents were involved in e-gambling and 26.8% of them could be classified as problem gamblers. The most popular e-gambling games were lotteries and sports betting. Gender, age, size of city of residence, level of education, and income were identified as significant predictors of involvement in e-gambling. The results indicated that men, younger people, and people who earnt less were more often involved in e-gambling. Having children, playing online scratch cards, and online sport betting—but not online lotteries—turned out to be typical for problem online gamblers. The prevalence of problem gambling among Polish e-gamblers suggests that extended research in this area is needed.

## 1. Introduction

The involvement of societies in gambling is a subject that has interested researchers for many years [1,2]. Gambling, as an entertainment form permitted for adults, involves a game in which there is a valuable stake, the result of which partially depends on chance, and it interests the representatives of social sciences mostly due to the potential damages that the activity can cause if specific circumstances arise. In 2013, in the 5th edition of the Diagnostic and Statistical Manual of Mental Disorders (DSM-5), pathological gambling was included in the group of non-substance related disorders (in section: Substance-Related and Addictive Disorders), which made the scientific world admit that pathological gambling and substance addiction have common development mechanisms and analogous symptoms [3]. The World Health Organisation included gambling disorder in the section “Disorders” due to substance use or addictive behaviours in the 11th edition of the International Classification of Diseases (ICD) classification. It is noteworthy that WHO distinguishes two categories of this disorder, including: 6C50.0 gambling disorder, predominantly offline, and 6C50.1 gambling disorder, predominantly online [4]. The development of addiction is most often described in four stages, common for substance and behavioural addictions. Taking the example of exercise addiction, they are as follows: recreational exercise, at-risk exercise, problematic exercise, and exercise addiction [5]. The traditional description of the gambling disorder has included four phases: the reaction to winning, losing, desperation, and hopelessness [6]. The next distinguished phases are accompanied by an increasing intensity of problems resulting from involvement in a given activity, progressive concentration and loss of control. According to researchers, the social consequences of gambling abuse include decreased productivity, social welfare costs resulting from absence from work [7], loss of jobs, early retirement, and even an increase in mortality resulting from suicidal tendencies occurring at advanced stages of gambling addiction [8,9]. Social detriments are also considered in the context of elevated distress and social isolation resulting from gambling [10].

Despite the long tradition of research on the involvement in gambling and problem gambling, a new phenomenon that has very quickly caused concern among the specialists emerged in the recent decades—online gambling [11]. Studies on online gambling have led many researchers to conclude that it has higher addictive potential than any other type of gambling [12,13,14,15,16]. Although results that do not confirm the higher addictive potential of online gambling do exist, most studies confirm this phenomenon [17,18]. Its high addictive potential is further confirmed by the higher rate of gambling addiction among online gamblers than among those who gamble in the traditional form [16,19,20]; this increase may even be three to five times higher [21]. The studies conducted by Effertz et al. on a representative group of 15,023 Germans showed that replacing 10% of offline gambling with online gambling increased the risk of becoming a problem gambler by 8.8–12.6% [22].

As for the increase in problem gambling among online players, the results of international studies conducted by McCormack among 1119 gamblers showed that 14% met the Problem Gambling Severity Index (PGSI) criteria for problem gambling, 29% met the criteria for moderate risk gambling, 32.7% met the criteria for problem gambling at a low level, and 24.3% did not show symptoms indicating problems resulting from gambling [23].

Why online gambling is more addictive remains uncertain; does it result from its higher accessibility, or, perhaps, from the nature of the Internet as a medium via which players gamble? [17,18]. Some studies have reported a lower percentage of addicted gamblers among “pure” online gamblers in comparison with “pure” offline gamblers [18]. Studies have, however, determined certain factors increasing the addictive potential of online gaming, including the games’ structure, which consists of, among other things, directness, accessibility, and ease of betting, all of which are particularly dangerous for young gamblers [24]. These factors are said by other gamblers, to have addictive potential. For example, Griffiths et al. also indicated factors related to the games’ structure—the directness of reinforcement, the speed of their course, and the frequency of game appearance—but also situational factors, such as accessibility and availability—which other researchers have also confirmed [25,26,27,28,29].

Online gambling seems to be more attractive for various reasons. It offers gamblers additional profits from gambling compared to offline gambling. First and foremost, gamblers can play whenever and wherever they choose (at home or work), which is associated with a high level of comfort and low access costs: players do not need to travel to certain locations, dedicate their time, and so forth [12]. Gamblers also save time because they can play several games simultaneously, which accelerates the course of online games [30,31]. The basic benefit of online gambling is anonymity, which seems to be particularly desirable for certain types of users [32,33].

Recent studies on involvement in online gambling have mainly focused on identifying the risk factors of problem gambling. The studies conducted by Effertz et al. on a representative group of Germans indicated that the risk of problem gambling decreases with higher levels of education and increases in the case of men; people who are unemployed, single, and divorced (respectively); and among migrants [22]. Effertz et al. also noticed that the risk of problem online gambling is the highest among heavy Internet users. Scientists have also drawn attention to the correlation between the type of game and problem gambling. For example, McCormack et al. observed that the risk of problem online gambling was significantly higher among people who gamble regularly and play online betting games, online slot machines, and online roulette, compared to gamblers who do not play regularly. In addition, persons who regularly played two or more types of online games were also at considerably greater risk of developing problem gambling than those who played one game only [23]. Other studies [34] have also highlighted the correlation between the number of the gambling accounts a gamer has, increased involvement in gambling, and increased intensity of problem gaming [35]. In the McCormack studies, people who regularly played only online poker were at lower risk of problem gambling than people who did not play poker regularly, but played other games as well, which is in line with the results of other studies [36]. Although the results suggest that there is a correlation between multi-gambling and problem gambling, researchers have emphasised the shortage of studies in the area [23].

There are few studies on gender differences in online gambling involvement. For example, studies conducted in Ireland have shown that women prefer online games that are more acceptable socially, such as lotteries or scratch cards [37]. Scratch cards are also the game type that women tend to become addicted to, which has also been highlighted by other researchers [32]. Women, on average, play for a shorter period of time (an average of two years for women and seven years for men), and spend less time gambling online than men (1 h per session, compared to 3 h for men).

Legal regulations are another aspect that has also been regarded as significant in recent studies on online gambling, particularly concerning the involvement of citizens in gambling. In his 2016 study, which included 1277 pathological gamblers undergoing addiction treatment, Chóliz discovered significant changes that occurred between 2012 (when online gambling was legalised in Spain) and the turn of 2014/2015. First and foremost, the number of people entering treatment due to pathological gambling in the studied facilities quadrupled. Also, most importantly, patients indicated online gambling as the source of their problems ten times more often than in 2012 (from 2.53% in 2012 to 24.21% in 2014/2015). For a comparison, the number of people indicating slot machines as the main source of their problems decreased (from 80.26% to 65.71%). These results seem particularly important in the context of the tendency to legalise online gambling and liberalise access to it, which has been observed by the specialists [38,39]. Moreover, researchers have also noted that using legal websites for online gambling causes less gambling-related damage [40], and a higher percentage of problem gambling occurs in populations in which legal regulations for online gambling are less restrictive [41].

The gambling market in Poland is regulated by the Act of 19 November 2009 on online gambling, which has so far been amended several times. In light of the Act of 15 December 2016 amending the Act on gambling, online gambling—with the exclusion of pari-mutuel betting and promotional lotteries—is subject to state monopoly [42]. Online games subject to state monopoly are organised by Totalizator Sportowy—a company owned by the State Treasury (Warsaw, Poland). The first online gambling games were introduced by Totalizator Sportowy in December 2018 and included a number of lotteries and games offered by the only legal online casino in Poland—slot machines, roulette, and card games for money. Legal online betting games are currently offered by nine private operators. Until 2017, when Poland tightened its restrictions on the betting market, introducing the possibility of blocking illegal domains, 90% of the industry belonged to the grey market [43].

Due to the relatively recent regulation of online gambling in Poland, there have thus far been no studies on the matter. It is worth noting that the longer tradition of research in Poland on addiction to slot machines resulted in the regulation of the market for these games [44]. The first study to estimate the involvement of Polish people in online gambling, and problem involvement in the activity and its determinants, was therefore, undertaken.

## 2. Materials and Methods

### 2.1. Participants

This study was conducted on a nationwide sample of 2000 adult Poles. The sample was representative and randomly selected on the basis of PESEL (Personal Identification Number). The distribution of gender, age, education, the size of place of residence, the region, and the number of people in the household were controlled. The Polish population was layered according to 9 geographical macro-regions, and then, in each of them, the localities were layered into 7 layers, according to size; the municipalities with probabilities proportional to the number of residents older than 18 years of age were randomly selected. Within the framework of each selected municipality, 6 interviews with randomly selected respondents were carried out. The respondents in municipalities were at first selected by means of drawing households proportionally to the number of household members according to the PESEL census records. Upon visiting a selected household, the interviewers selected the respondents using the Kish grid and then conducted computer-assisted personal interviews with them. If conducting the interview with the person from the list was not possible, the interviewer would look for a person of the same age and gender in the same town. The study was carried out by the GfK Polonia, the Polish branch of a well-known international public opinion research institute (GfK SE, Nuremberg, Germany), which also ensured the anonymity and uniformity of testing.

### 2.2. Measures

#### 2.2.1. E-Gambling

The questionnaire consisted of questions regarding use of online gambling games, frequency, frequency of online gambling, time duration of a single session, and money spent on online gambling. In the first question, the respondent was asked to select the online games on which they have bet money at least once within the last 12 months. The list included 11 categories: online lottery (i.e., receipt lottery), online scratch cards, slot machines and other gambling machines on the Internet, online card games for money, other online casino games (i.e., roulette, dice), Totalizator Sportowy number games via the Internet, other online number games (i.e., bingo), online arcade games for money, online sports betting (including “fantasy sports”), online betting on e-sport or online virtual sports, and online betting on financial markets (i.e., stock exchange, FOREX, binary options). A memory-activating filtering statement was posed: “I am certain that I have not made online cash bets within the last 12 months.” The question regarding e-gambling frequency was: How often, within the last 12 months, have you gambled online? (1 = everyday; 2 = several times a week, but not every day; 3 = once a week; 4 = several times a month but more rarely than once a week; 5 = once a month; 6 = several times a year, but more rarely than once a month). The question regarding the time duration of a single session was: How much time did one session of the game usually take? (1 = less than 15 min; 2 = from 15 to 30 min; 3 = from 31 min to an hour; 4 = from over an hour to 2 h; 5 = from over 2 h to 3 h; 6 = more than 3 h). The question regarding spending was open: “How much money exactly have you spent on online gambling within the last 4 months?”

#### 2.2.2. Problem E-Gambling

The Brief Biosocial Gambling Screen (BBGS), adapted to Polish by Niewiadomska et al., was adjusted to the assessment of problem e-gambling [45,46]. It contains three questions about gambling and has been shown to have good screening properties for the criteria of problem gambling compliant with DSM-5 [47]. Each of the questions could be answered with either “Yes” or “No.” The risk of problem gambling cut-off at endorsing one symptom is the best indicator of gambling disorder, taking into account that sensitivity and negative predictive value are most important for identifying individuals who potentially need treatment [45,47]. The psychometric properties of the Polish adaptation of BBGS were tested on a representative sample of high school students of the Lublin province. The criterion was fulfilment of at least 5 DSM-IV criteria of pathological hazard, measured by self-report. Sensitivity was 0.82, and specificity was 0.96 [46]. Because not distinguishing by a measure of problem gambling between forms of gambling (online versus offline) is a potentially confounding issue [48], we adjusted the BBGS for the purposes of the current study by rewording the questions in the BBGS to refer to online gambling. The final versions of the questions used in this study were as follows: Within the last 12 months, have you felt powerless, irritated, or anxious when you were trying to quit or limit online gambling? Within the last 12 months, have you tried to keep the fact that you are gambling online from your family and friends? Have you had financial trouble resulting from online gaming, because of which you had to ask your family, friends, or social services for financial support within the last 12 months? 

#### 2.2.3. Sociodemographic Variables

Information on the sociodemographic profiles of the respondents was obtained from data collected by the interviewer and from the questionnaire. The sociodemographic questions were answered by 2000 people. Their average age was 45.61 years (SD = 18.456, minimum = 18, maximum = 94). Table 1 presents the sociodemographic descriptive statistics.

### 2.3. Data Analysis

All statistical analyses were performed using IBM SPSS version 24 software [49]. We used methods and statistics appropriate to the types of measurement scale and the specific parameters applied. Categorical data are presented as frequencies and percentages, and group comparisons were made using the χ^2^-test. We used logistic regression (enter method and simple contras, with the reference category first) to identify the variables that best predict involvement in e-gambling.

## 3. Results

### 3.1. Popularity of Gambling and Types of Games

The results obtained indicate that 83 (4.1%; 95% CI (3.3%, 5.1%)) of the 2000 respondents surveyed in the last 12 months have made monetary bets using online gambling services. Most respondents played Totalizator Sportowy lotteries, online sports betting, sports betting concerning e-sport or virtual sport, and online card games for money. Online slot and gambling machines and online betting on the financial markets were used least frequently. None of the respondents indicated using online arcade games for money (Table 2).

### 3.2. Sociodemographic Variables Associated with Online Gambling

Logistic regression was used to assess the factors associated with involvement in online gambling (cf. Table 3). The dependent variable had two categories (1—gambling versus 0—not using online gambling services in the last twelve months). Age, gender, population of the place of residence, education, income level, having children, and frequency of Internet use were considered as independent variables. The factors that significantly explained involvement in online gambling were: gender, age, population of the place of residence, education, and monthly family income. Men were more likely to be involved in gambling activities than women. In terms of age, the youngest group (up to age 29) was significantly more likely to be involved in online gambling than older people (over 50). Analysing the size of the place of residence, people living in the countryside were significantly different from those living in towns with 20,000–100,000 residents and those coming from cities of 200,000–500,000 residents. Online gambling activity was much less frequent among people living in towns or cities compared to people living in the countryside. Higher online gambling activity could also be seen among people with primary education compared to those with vocational education. Monthly income was also an important factor explaining involvement in online gambling. People with low monthly incomes were much more likely to devote their time to online gambling than those earning more than PLN 3000. Frequency of Internet use was also an element co-existing with online gambling activity. Individuals using the Internet more frequently were also inclined to become more involved in online gambling.

### 3.3. Prevalence of Problem Gambling among Players and Related Factors

Out of the 83 people who participated in online gambling, 22 (26.8%; 95% CI (17.9%, 36.7%)) were at risk of becoming problem gamblers. This group consisted of respondents who provided at least one affirmative answer on the BBGS scale. In order to determine the characteristics of problem online gamblers, we compared them to non-problematic gamblers in respect of sociodemographic variables using the chi-square test (cf. Table 4).

A comparison between gamblers at risk of becoming problem gamblers and those who were not at such risk indicated several differentiating variables (cf. Table 4). These included having children and choice of online e-gambling services. Individuals who manifested symptoms of problem use of e-gambling more frequently had children than those with no symptoms of problem gambling. PeGs were also less active in playing Totalizator Sportowy lotteries and more often used online sports betting (including fantasy sports) and online scratch cards.

## 4. Discussion

The results of our study allowed us to determine the extent of the involvement of adult Poles in online gambling. First, it is worth mentioning that the first legitimate online gambling games in Poland were sports betting services, first organised in 2012. The provision of other online gambling services was regulated only by the 2016 Act, under which the provision of other online games is subject to a state monopoly [42]. It should be noted that, in practice, these games were made available on the market only in December 2018, which highlights the specificity of the e-gambling market in Poland and sheds light on the results of this study, which was conducted in December 2018. The lotteries organised by Totalizator Sportowy proved to be the most popular online and offline gambling games, having been the most common type of such games for Poles [50]. It is worth noting here that these games, covered by the state monopoly, may be advertised in public media, which significantly increases their potential accessibility compared to other types of gambling games, the advertising of which is prohibited by law. The second most popular games included sports betting and betting related to e-sports and virtual sports, the popularity of which may be explained by the relatively long history of this type of online gambling in Poland. The popularity of gambling reflects, to a large extent, the cultural specificity or legal regulations of a given country concerning the availability of games. For example, the most popular online gambling game in France is, among others, horse race betting, which illustrates the long-standing tradition of what is considered to be a national sport in France [51]. In Poland, football plays a similar role. The relatively high interest of Poles in e-sports or online virtual sports betting is a new trend. This phenomenon has not yet been assessed, so it is difficult to estimate the extent to which there is an upward trend or whether the behaviour has been long-standing. Taking into account the novelty of the phenomenon of e-sports betting, not only in Poland but also worldwide (e.g., this phenomenon was included in the national survey “e-Games France 2017” for the first time only in 2017), an upward trend may be expected [51]. The interest of Poles in such betting even exceeds the popularity of online card games, which is fourth in terms of popularity. The interest in online gambling as a whole seems to be low in Poland. During the 12-month period prior to the survey, 4.1% of adult Poles made an online bet, which is a very small percentage compared to the 37.1% of Poles engaged in offline gambling during the same period [48]. It is worth mentioning that, in the latest epidemiological study on behavioural addictions in Poland, only 1.2% of Poles declared that they had gambled online in the past year. However, it is also significant that, in this study, the category of online gambling was only one among nine categories of offline gambling.

Due to the fact that this was the first study on e-gambling in Poland, we were interested in determining which individuals choose this form of entertainment most often. The results showed that e-gambling was more popular with men than women, and that interest decreases with age. These data are confirmed by studies conducted in other countries [26,37]. Additionally, online gambling was more popular among those with incomes lower than the national average salary than those with incomes equal to or higher than the national average wage. Involvement in online gambling also decreased with the decline in daily Internet use. This phenomenon was all the more alarming because, in light of the Effertz study, the risk of problem online gambling is largest among highly engaged Internet users [22], which was confirmed by the research of Rémond and Romo [52]. The results obtained are in line with other studies, which show that the popularity of online gambling in the West is attributable, among other things, to low access costs—the gambler does not need to travel to the place where the game is played or devote time to such travel [12]. This makes online gambling more accessible from an economic point of view, and therefore more likely to be selected by people with lower socioeconomic status. Other researchers have also emphasised that accessibility and availability are the factors contributing to increased involvement in online gambling [25,26,27,28,29].

Finally, we were interested in the extent to which problem gambling was exacerbated among online gamblers. The study results showed that 26.8% of gamblers had symptoms indicating a probable gambling addiction on the basis of the BBGS scale. These results reveal that the risk of gambling addiction is higher among Polish online gamblers than among gamblers in general (both online and offline). In light of the latest results of a national survey conducted by the Centre for Public Opinion Research (CBOS: Centrum Badania Opinii Społecznej) on behavioural addictions, 7.7% of all gamblers show a low addiction risk, 0.9% of them a moderate risk level and 0.9% of gamblers are problem gamblers, making a total of 9.5% of gamblers at risk of addiction [53]. It should be mentioned, however, that the CBOS survey was conducted using the Canadian Problem Gambling Index (CPGI) scale.

Relating the results obtained in our study to other studies, a convergence may be observed. International online gambling studies have showed, for instance, that 14% of gamblers met the criteria for problem gambling in accordance with the Problem Gambling Severity Index, 29% met the criteria for risky gambling and 32.7% of gamblers met the criteria for problem gambling at a low level. Other studies also confirm greater exacerbation of problem gambling among online than offline gamblers [16,19,20], which is, according to some, even three to five times greater [21]. In Austrian studies, 31% of online gamblers showed symptoms of problem gambling, while 18% of offline gamblers displayed such symptoms in accordance with Lie-Bet questionnaire results [54].

The last aspect we analysed included sociodemographic variables coexisting with gambling addiction.

The first important factors differentiating gamblers at risk of a gambling disorder from non-problem gamblers were male gender and age from 40 to 49 years old. These results are surprising because Poles addicted to offline gambling are mainly younger people (18–24 years old) [53]. Nevertheless, the results obtained by us are consistent with the Austrian research of Yazdi and Katzian, in the light of which addicted online gamblers most often belong to the age range 30–49 years [54]. It seems that due to the relatively short period of online gambling being available in Poland, these games are mainly used by mature men who have longer experience with offline gambling, and are, therefore, consciously looking for a new offer of already known entertainment.

The level of education also differentiated problem and non-problem gamblers. Problem e-gamblers more often have primary education, which is also the case with offline gamblers in Poland.

The next important factor differentiating gamblers at risk of a gambling disorder from non-problem gamblers was the presence of children in the household. Gamblers at risk constituted a group that more often had children than representatives of the non-addicted group. This may be due to the higher age of problem e-gamblers. At this stage of study, we are still considering how to understand this relationship. It may be argued that people who are interested in gambling—and have children—have been more inclined to opt for online games which are more accessible due to time constraints. As studies confirm the stronger addictive potential of online gambling, this activity is, thus, more likely to turn into addiction. It can also be assumed that gamblers who do not have children gamble offline as well, but also opt for alternative, non-addictive offline entertainment, which is less accessible to those with children. Online gambling is more absorbing, allowing the gambler to play several games at once, as emphasised by both Cotte et al. and Gainsbury et al. [30,31]. The structural characteristics of online games—such as directness, accessibility, ease of betting, and the fact that they pose a particular risk of addiction—are also highlighted by Chóliz [24].

Accessibility factors are also revealed when linking residence to problem gambling. In light of the results, problem e-gamblers come, more often, from the countryside. Despite the fact that rural residents do not gamble online more often than urban residents, they develop problem e-gambling more often. It can be assumed that these people, with limited possibilities of enjoying other entertainment (including offline gambling), engage in e-gambling more intensively, which translates into an increased risk of developing addiction to these games.

Another factor significantly co-existing with the risk of gambling addiction was the type of game being played. In light of the results, gamblers at risk of addiction are more often involved in sports betting (including “fantasy sports”). These results correlate with McCormack’s study, which showed that the risk of problem online gambling is significantly higher for, among others, online sports betting gamblers [23]. Given that sports betting is one of the most popular online gambling services in Poland, this is an important discovery. Problem e-gamblers are also more often involved in online scratch cards, which are one the most popular offline gambling game types in Poland [53].

Summarising the results of the study, it is worth once again referring to the Polish legal regulations on gambling. When the study was conducted, legitimate gambling, apart from sports betting, was in its early stages. It would be significant to monitor the development of Poles’ involvement in online gambling as it becomes more widespread. Taking into account the relatively low involvement of Poles in online gambling, it may be assumed that the results obtained stem from the fact that these games were not yet very popular at the time the study was conducted. For instance, Chóliz [24] analysed the changes that occurred between 2012 (when online gambling was legalised in Spain) and the turn of 2014/2015, during which period the number of people who started treatment for pathological gambling quadrupled. Additionally, patients indicated online gambling as the main source of their problems nearly ten times more often in 2014/2015 than in 2012. With this in mind, it is extremely important to continue epidemiological studies on participation in online gambling and problem gambling to develop recommendations for legislators based on changes in the behaviour of gamblers resulting from the implementation of legal amendments. Legalisation of online gambling is a very important issue. Researchers note that the use of legitimate gambling sites causes less gambling-related harm than the use of illegal sites [40]. However, the legalisation of online gambling alone cannot be the only preventive factor. Its effects should be monitored and the next steps need to be adapted accordingly. It would be worthwhile to conduct future studies on the relationship between multi-gambling and gambling problems, especially as researchers have pointed out a research deficit in this area [23]. It would also be important to highlight the differences between “pure” online gamblers, “pure” offline gamblers, and “mixed-mode” gamblers. It is also significant to recognise the differences between the genders in terms of online gambling activities. As the online gambling market in Poland is constantly evolving, it is important to use the experience of Western countries when implementing responsible gambling policy. Internet gamblers should be informed by operators about the risks of gambling. In addition, it would be important for operators to monitor online gambler behaviour and identify at risk gamblers and direct messages to them about threats and the possibilities of seeking help. Gamblers should be able to exclude themselves from the site for a certain period of time, and this should also be offered to at risk gamblers. Some activities in this area are already being implemented; however, it is important to conduct research on the effectiveness of preventive measures taken, because cultural factors can modify it.

### Limitations

Despite the pioneering character of this study in Poland, this study also has its limitations. The first one is the BBGS, the research tool used, which, despite its psychometric properties, only has a screening character and is used relatively rarely in epidemiological studies. Earlier studies on offline gambling in Poland employed a different scale (CPGI), so it is difficult to compare those results with the results of the present study. The BBGS was used due to the preliminary nature of this study, the continuation of which is being planned. Besides, the adjustment of BBGS to online gambling by rewording the questions, being our attempt to remedy to the lack of distinction between online and offline forms of gambling, is a very unusual technique, and it is uncertain how that step affected the results obtained. The next limitation was the restricted number of questions in the survey and the resulting failure to include more variables, including psychological ones. A more elaborate study is, however, currently underway. There were also no questions about offline gambling in the survey, which makes it impossible to determine whether the outspoken gamblers are “pure” online gamblers. In light of the study by Gainsbury, there are differences between pure online and offline gamblers and “mixed-mode” gamblers [18]. Another limitation of research is the fact that it was conducted in the same month in which the online gambling market was expanded. The result of this may have been that the study only captured gamblers very advanced in using new technologies who were the first to reach games in a new form. This confirms the connection between the use of online gambling games and the intensity of Internet involvement. Another hypothesis may be that the research revealed players looking for new types of gambling. This hypothesis, however, is partly undermined by the results of studies in the light of which most Poles practiced Lottery of Sports Totalizator and online sports betting. Lotteries are the most popular offline games in Poland; they are widely recognized and their publicity is allowed, which translates into their high availability, and therefore—greater involvement of Poles in them. On the other hand, online sports betting has been available in Poland since 2012, which is why it is not a “new” type of game. Research should certainly be continued to learn more about the specifics of Poles’ involvement in online gambling.

## 5. Conclusions

This study provided a characterisation of Poles’ involvement in online gambling. This is all the more relevant because in December 2018 new types of online gambling services were introduced. We thus managed to capture the ‘initial’ state—that is, the very beginning of the new reality of online gambling in Poland. As a result, it is possible to observe changes arising from the introduction of new legal regulations. Studies have shown that 4.1% of Poles made an online bet in the 12-monthnperiod prior to the survey. Lotteries, sports and e-sports betting proved to be the most popular online gambling games. Online gambling was more popular among younger men, with incomes lower than the national average salary, who were highly engaged Internet users. Among all gamblers, 26.8% were reported to be at risk of gambling addiction based on the results of the BBGS screening questionnaire. Addicted online gamblers more often had children and preferred sports betting.

## Figures and Tables

**Table 1:** Descriptive statistics for the sociodemographic variables for the test group (*n* = 2000).

Variable	Category	*n*	%
Gender	Male	964	48.2
	Female	1036	51.8
Place of residence	countryside	815	40.8
	town of up to 20,000 residents	263	13.1
	town of 20,000–50,000 residents	213	10.7
	town of 50,000–100,000 residents	163	8.2
	town of 100,000–00,000 residents	151	7.6
	city of 200,000–500,000 residents	182	9.1
	city of more than 500,000 residents	212	10.6
Education	primary	481	24.0
	basic vocational	469	23.4
	secondary	683	34.1
	higher	368	18.4
Frequency of Internet use	nearly every day	1209	60.5
	at least once in a month	276	13.8
	less frequently or does not use at all.	514	25.7
Monthly household income	up to PLN 2000	156	7.8
	PLN 2000–2999	231	11.5
	PLN 3000–4499	487	24.4
	PLN 4500 and above	1125	56.3
Children living in the same household	Yes	268	63.4
	No	732	36.6

Note: PLN—Polish zloty; 1€ ≈ 4.3 PLN.

**Table 2:** Prevalence of forms of online gambling (*N* = 2000).

Game Type	*n*	%	95% CILL	95% CIUL
Totalizator Sportowy lotteries	41	2.0	1.5	2.7
Online sports betting (including “fantasy sports”)	19	1.0	0.6	1.4
Online betting on e-sports or online virtual sports	11	0.6	0.3	0.9
Online card games for money	10	0.5	0.3	0.9
Online scratch cards	6	0.3	0.1	0.6
Other online casino games (e.g., roulette, dice)	5	0.2	0.1	0.5
Internet lottery (e.g., receipt lottery)	4	0.2	0.1	0.5
Other online lotteries (e.g., bingo)	4	0.2	0.1	0.5
Slot machines or other online gambling machines	1	0.1	0.0	0.2
Online betting on financial markets	1	0.1	0.0	0.2
Online arcade games for money	0	0.0	0.0	0.0

Note: 95% CILL: 95% confidence interval lower limit; 95% CIUL: 95% confidence interval upper limit.

**Table 3:** An explanatory model of e-gambling (*n* = 2000).

Variables	B	SE	Wald	df	*p*	Exp(B)	95% CI for EXP(B)	
							Lower	Upper
Gender: (ref.: male)	−1.129	0.282	16.055	1	<0.001	0.323	0.186	0.562
Age: (ref.: 15–29 years of age)			21.002	5	0.001			
30–39	−0.527	0.367	2.067	1	0.150	0.590	0.288	1.211
40–49	0.414	0.319	1.685	1	0.194	1.513	0.810	2.829
50–59	−2.904	1.004	8.366	1	0.004	0.055	0.008	0.392
60–69	−1.392	0.681	4.178	1	0.041	0.249	0.065	0.944
70 and above	−2.135	1.166	3.353	1	0.067	0.118	0.012	1.162
Population of the place of residence: (ref.: countryside)			15.507	6	0.017			
town of up to 20,000 residents	0.072	0.377	0.036	1	0.849	1.074	0.513	2.248
town of 20,000–50,000 residents	−1.266	0.559	5.125	1	0.024	0.282	0.094	0.844
town of 50,000–100,000 residents	−1.103	0.580	3.615	1	0.057	0.332	0.106	1.035
town of 100,000–200,000 residents	−0.649	0.509	1.625	1	0.202	0.523	0.193	1.417
city of 200,000–500,000 residents	−4.465	2.268	3.876	1	0.049	0.012	0.000	0.980
city of more than 500,000 residents	0.307	0.379	0.659	1	0.417	1.360	0.647	2.855
Education: (ref.: primary)			14.184	3	0.003			
basic vocational	−1.851	0.547	11.450	1	0.001	0.157	0.054	0.459
secondary	−0.189	0.327	0.335	1	0.563	0.828	0.436	1.570
higher	0.244	0.378	0.415	1	0.519	1.276	0.608	2.675
Children living in the same household: [ref.: no children]	0.260	0.289	0.809	1	0.368	1.297	0.736	2.283
Household net income: (ref.: up to PLN 2000)			24.876	3	0.000			
PLN 2000–2999	−1.105	0.580	3.630	1	0.057	0.331	0.106	1.032
PLN 3000–4499	−1.732	0.532	10.593	1	0.001	0.177	0.062	0.502
PLN 4500 and above	−2.405	0.517	21.649	1	0.000	0.090	0.033	0.249
Internet use: (ref.: nearly every day)			5.077	2	0.079			
at least once in a month	−0.128	0.481	0.071	1	0.789	0.880	0.343	2.256
less frequently or does not use at all	−1.758	0.782	5.054	1	0.025	0.172	0.037	0.798
constantly	−4.682	0.450	108.157	1	0.000	0.009		

Note: Overall model evaluation: Likelihood ratio test: χ^2^(21) = 167.916; *p* < 0.001; Cox and Snell R^2^ = 0.081; Nagelkerke’s R^2^ = 0.276.

**Table 4:** Comparison of sociodemographic variables between non-problem online gamblers (NPeG, *n* = 61) and problem online gamblers (PeG, *n* = 22).

Variables	NPeG		PeG		χ^2^	*p*
	*n*	%	*n*	%		
Gender:					0.381	0.537
Male	45	75	15	68.2		
Female	15	25	7	31.8		
Age:					8.998	0.109
15–29	34	55.7	6	28.6		
30–39	10	16.4	3	14.3		
40–49	14	23.0	10	47.6		
50–59	1	1.6	0	0		
60–69	1	1.6	2	9.5		
70 and above	1	1.6	0	0		
Population of the place of residence:					5.320	0.503
countryside	25	41.7	14	60.9		
town of up to 20,000 residents	9	15.0	2	8.7		
town of 20,000–50,000 residents	3	5.0	1	4.3		
town of 50,000–100,000 residents	4	6.7	0	0		
town of 100,000–200,000 residents	5	8.3	0	0		
city of 200,000–500,000 residents	0	0	0	0		
city of more than 500,000 residents	14	23.3	6	26.1		
Education:					4.871	0.181
primary	16	26.2	11	50.0		
basic vocational	4	6.6	1	4.5		
secondary	22	36.1	7	31.8		
higher	19	31.1	3	13.6		
Children living in the same household:	24	40.0	15	68.2	5.126	0.022
Household net income:					3.540	0.316
up to PLN 2000	7	11.7	4	19.0		
PLN 2000–2999	8	13.3	1	4.8		
PLN 3000–4499	20	33.3	4	19.0		
PLN 4500 and above	25	41.7	12	57.1		
Internet use:					1.151	0.562
nearly every day	52	85.2	20	90.9		
at least once in a month	6	9.8	2	9.1		
less frequently or does not use at all.	3	4.9	0	0		
Types of online gambling services:						
Internet lottery	2	3.3	2	9.1	1.150	0.284
Online scratch cards	2	3.3	3	13.6	2.984	0.084
Slot machines or other online gambling machines	1	1.7	0	0	0.371	0.542
Online card games for money	8	13.1	2	9.1	0.247	0.619
Other online casino games (e.g., roulette, dice)	5	8.2	0	0	1.919	0.166
Totalizator Sportowy lotteries	36	59.0	5	21.7	9.289	0.002
Other online lotteries (e.g., bingo)	3	5.0	1	4.5	0.007	0.933
Online arcade games for money	0	0	0	0.0	-	-
Online sports betting (including “fantasy sports”)	11	18.3	8	36.4	2.940	0.086
Online betting on e-sports or online virtual sports	10	16.7	1	4.5	2.036	0.154
Online betting on financial markets	1	1.6	0	0	0.365	0.546

Note: PeG—problem online gamblers; NPeG—non-problem online gamblers.
